# Characterization of a 10 W class electrospray array thruster

**DOI:** 10.1007/s44205-025-00114-4

**Published:** 2025-03-26

**Authors:** Collin Whittaker, Steven Arestie, Colleen Marrese-Reading, Benjamin Jorns

**Affiliations:** 1 Department of Aerospace Engineering, University of Michigan, City, 48109 Michigan United States; 2 NASA Jet Propulsion Laboratory, California Institute of Technology, Pasadena, 91109 California United States

**Keywords:** Electrospray, Micropropulsion, Electrohydrodynamics, ILIS

## Abstract

A porous conical type electrospray array thruster consisting of 6102 individual emitters is operated at up to 13.3 W power. The design and manufacture of the thruster are described, including its porous glass emitter chip and metallized ceramic extractor chip. A precision mass balance mounted inside a bell jar is used to directly measure the thrust, specific impulse, and efficiency in negative polarity, from $42\pm 0.5\ \upmu$N, $1050\pm 26$ s, and $57\pm 1.9$% at $-1000$ V and 0.38 W to $174\pm 0.5\ \upmu$N, $420\pm 2$ s, and $21\pm 0.3$% at $-1300$ V and 1.7 W. Additional negative polarity experiments in a 2 meter vacuum facility demonstrate powers from order 1 $\upmu$W to over 10 W, spanning 7 orders of magnitude. Power and performance measurements were not repeated for positive mode operation, as this was found to induce arcing between the emitter and extractor electrodes at 1400 V and above. The drop in efficiency from $-1000$ V to $-1300$ V operation in the bell jar is discussed within the context of facility effects, and secondary charged particle flux to the thruster is identified as a likely contributor. Finally, the performance of the thruster is considered relative to scaling electrospray systems to higher power robustly.

**Supplementary Information:**

The online version contains supplementary material available at 10.1007/s44205-025-00114-4.

## Introduction

Electrospray array thrusters are a potentially enabling technology for small spacecraft because they can access low power regimes ($<100$ W) where there are few feasible propulsion options. Moreover, porous architecture thrusters operating on ionic liquids could have very low mass and high efficiency for a given thrust and power [1], which opens up novel rapid-transit missions in cislunar space [2]. However, the low thrust typically produced by individual electrospray emitters requires that many of them be aggregated together to achieve higher thrust or that wedge geometries be adopted to promote the formation of additional beamlets [3–5], forming an electrospray array thruster [6–10].

At the system level, one strategy for increasing size and power is by tiling several electrospray array thrusters together [7, 11]. This has the advantage of providing potential fault tolerance and greater modularity through redundant thruster heads, but it increases the complexity of the power processing unit and limits specific mass by duplicating support structures that do not contribute to thrust (e.g. electrical insulation, fixtures). Alternatively, scaling in power by increasing the number of emitters in a single array could decrease the specific mass with increasing power by avoiding these parasitic structures. However, this makes the system more prone to failure by electrical short [12], and presents challenges in maintaining tolerances in emitter fabrication and alignment when extending manufacturing methods. These disadvantages are partially mitigated by implementing a resilient extractor architecture that provides fault tolerance [13, 14]. That said, fully accounting for emitter variability and poorly understood physical phenomena to maintain thruster performance requires detailed manufacturing characterization [15], rigorous uncertainty quantification [16–18], and robust design methodologies [19].

These techniques do not wholly eliminate the need to physically realize and experimentally characterize larger-scale electrospray systems, which both inform how manufacturing processes scale to higher power systems and provide data with which to train predictive design models. Indeed, to the authors’ knowledge no monolithic porous electrospray array thruster has been reported in the literature with more than 1000 emitters. To that end, we present the design and characterization of a 10 W-class porous conical type electrospray array thruster. In the Methodology section, we describe our methodologies, including the design and manufacture of the thruster and the experimental facilities where we conducted tests. Then, in the Results section, we detail the results of our experiments, including direct performance measurements of the system at low power and expanded throttling to high power. We next discuss our results in the Discussion section, interpreting them physically and considering them within the context of continued electrospray thruster development and scaling.

## Methodology

In this section, we describe the methodologies underlying our work, including the design and manufacture of the thruster and the experimental facilities used to characterize it.

### Thruster design and manufacturing

**Fig. 1:**
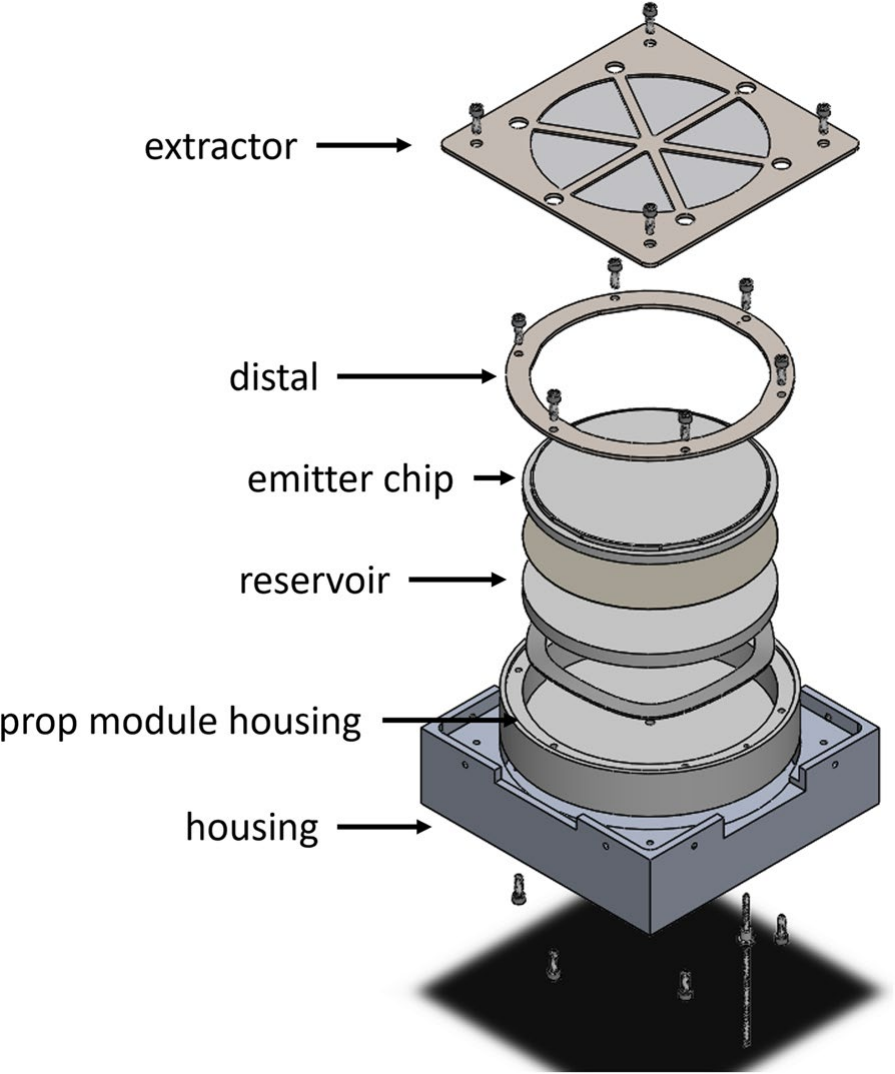
Exploded view of the MEAT-1.2 system, with key components labeled

The thruster characterized in this work is the Michigan Electrospray Array Thruster Series 1 Version 2 (MEAT-1.2). We show an exploded diagram of the system in Fig. 1.

The MEAT-1.2 is a porous conical-type electrospray array thruster, and it takes design heritage from the Air Force Electrospray Thruster (AFET) family of systems [8]. At the center of the thruster is an emitter module which contains the propellant-wetted components, including the emitter chip and a porous reservoir. An extraction electrode sits above the emitter module, with the emitter module and extractor situated inside a larger housing. This main thruster housing is 88.9 mm $\times$ 88.9 mm $\times$ 25.4 mm and made of aluminum; it provides fixture points for the emitter module and extractor, alongside set screws to adjust the alignment of the two components. The MEAT-1.2 platform can accommodate multiple different emitter and extractor chip designs (varying emitter size, etc.); the emitter and extractor chip designs used here have serial designation 2-02.

#### Emitter chip

**Fig. 2:**
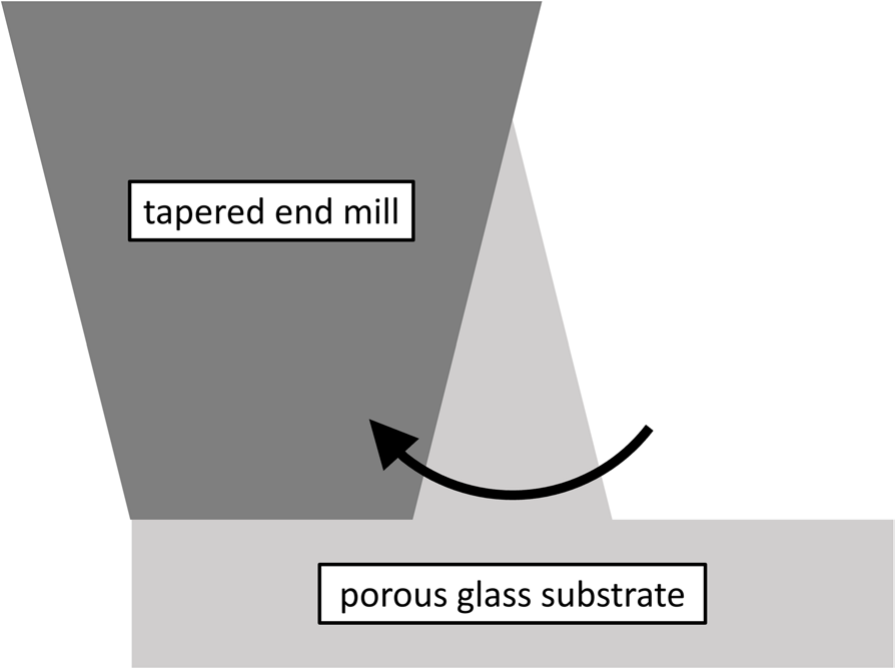
Schematic of emitter machining operation

The emitter chip is fabricated from a 70 mm diameter P5 grade (1 $\upmu$m nominal pore size) sintered borosilicate glass frit. The emitter structures are conical, and we machine them from the substrate using a miniature square tapered end mill on a computer numerical control (CNC) milling machine. The CNC mill is programmed to make a series of circular cuts at full machining depth to remove material, leaving the emitters in relief at the center of the cuts—the taper in the end mill produces the inverse taper in the emitters. We show this schematically in Fig. 2.

The height and basal radius of the emitters is controlled by the depth and radius of the cutting path but must be offset by the diameter of the cutting tool and runout in the spindle of the CNC mill (i.e., precession of the cutting tool about the rotational axis). The tool diameter and runout also limit the pitch and packing density of the emitters. The emitter chip presented here consists of 6102 emitters of maximum height 455 $\upmu$m packed in a hexagonal tiling with a 660 $\upmu$m pitch.

The abrasive nature of the cutting operation produces wear on the tools. To mitigate this wear, we use tools coated in diamond by chemical vapor deposition (CVD) and douse the tool in a jet of lubricating coolant—an emulsion of oil in water—during machining. Nevertheless, to ensure sufficient tool life to complete the chip, we divide the cutting operations between two separate end mills (whose diameter and runout must be compensated for individually on account of manufacturing variance between tools). Additionally, during machining the coolant infiltrates the porous medium, contaminating it with nonvolatile oils; it is therefore necessary to clean the chip after machining, which we accomplish by first immersing the chip in a series of baths of polar and nonpolar solvents and then forcing a detergent through the chip under pressure.

The finished emitter chip is coupled to a porous reservoir—a 70 mm diameter P4 grade (10 $\upmu$m nominal pore size) borosilicate glass frit—through a cellulose filter paper (also 10 $\upmu$m pore size). This stack of components is compressed by a stainless steel split wave disc spring and retained within a polyether ether ketone (PEEK) housing by a laser-cut stainless steel ring, which also serves as a distal electrode for the system. A series of flats machined around the sides of the emitter chip serve to align it to the distal electrode and hence the thruster body.

#### Extractor chip

The extractor chip consists of a metallized ceramic bonded into a support frame. It contains a series of circular apertures that are matched to the pattern of the emitter chip. Two sheets of MACOR ceramic each approximately 75 mm square are first faced on a CNC mill to be flat within the machine reference frame. We then laminate the two sheets together using a low-viscosity ($<5$ cP) cyanoacrylate adhesive. The top sheet is then further faced on a CNC mill to achieve a desired thickness. For the extractor chip here, the chip was machined to be 455 $\upmu$m thick.

Once faced to the desired thickness, the exposed surface of the top sheet is metallized with silver using a DC magnetron sputtering system. The sputterer was operated at a current of 30 mA for 540 seconds, producing a metal film with an average sheet resistance of order 10 $\Omega$/sq. The coated laminate is then returned to the CNC mill and apertures are drilled clean through the top sheet and into the bottom sacrificial sheet using a miniature spotting drill coated in CVD diamond. For this chip, the apertures are nominally 440 $\upmu$m in diameter. After all 6102 apertures are machined, an end mill is used to cut the profile of the chip body and the bonded sheets are transferred to an acetone bath to delaminate.

**Fig. 3:**
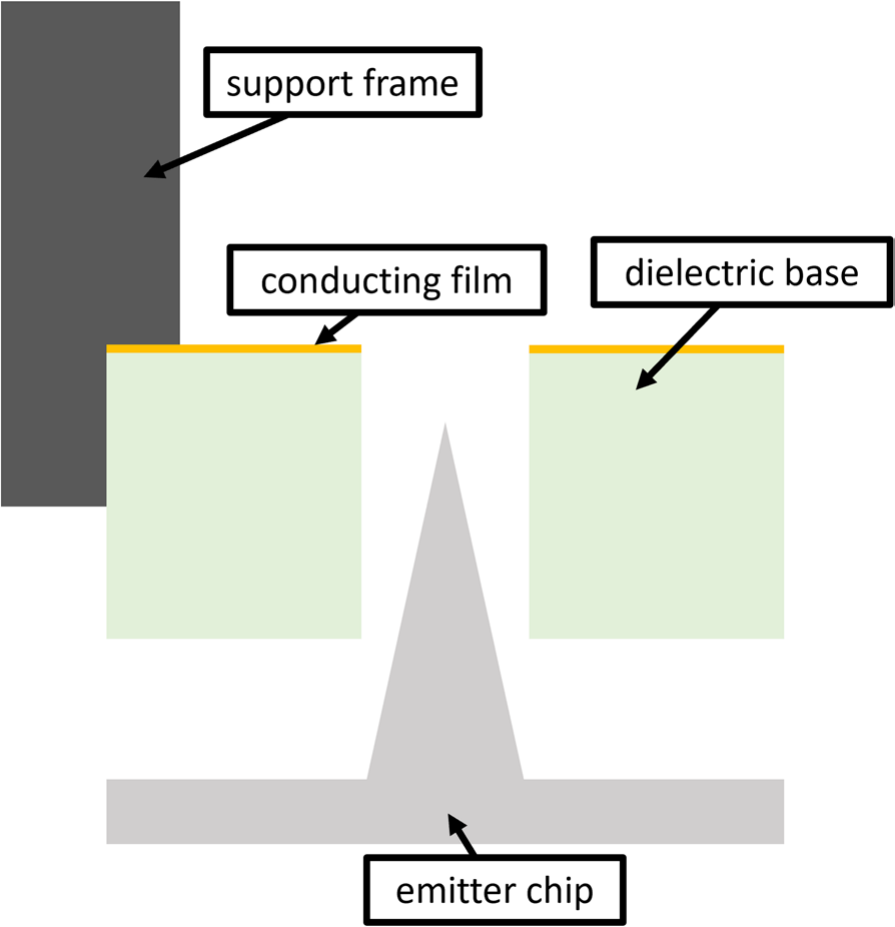
Relative orientation of emitter and extractor chips

In parallel, a sheet of stainless steel is laser cut to serve as a structural support frame for the extractor chip. A pocket of depth 250 $\upmu$m is then machined in the face of the frame, designed to match the profile of the emitter chip (including flats for alignment). The frame features struts across the width of the chip (see Fig. 1) that serve to reduce flexure. These struts shadow locations where emitters and extractor apertures would otherwise appear, so these sites are programmatically excluded during the emitter and extractor cutting processes. Finally, the metallized side of the delaminated extractor chip is bonded into the retaining pocket of the frame with a low outgassing conductive epoxy. In this way, the metallized face of the extractor chip is on the downstream side relative to the emitters, an orientation we show in Fig. 3.

### Experimental facilities

We conducted our experiments in the NASA Jet Propulsion Laboratory’s MicroPropulsion Laboratory (NASA JPL MPL), an ISO Class 4 cleanroom equipped with metrology tools (e.g., microscopes) to assist with thruster fabrication and vacuum facilities of various scale and diagnostic apparatuses to support thruster testing.

#### Bell jar

**Fig. 4:**
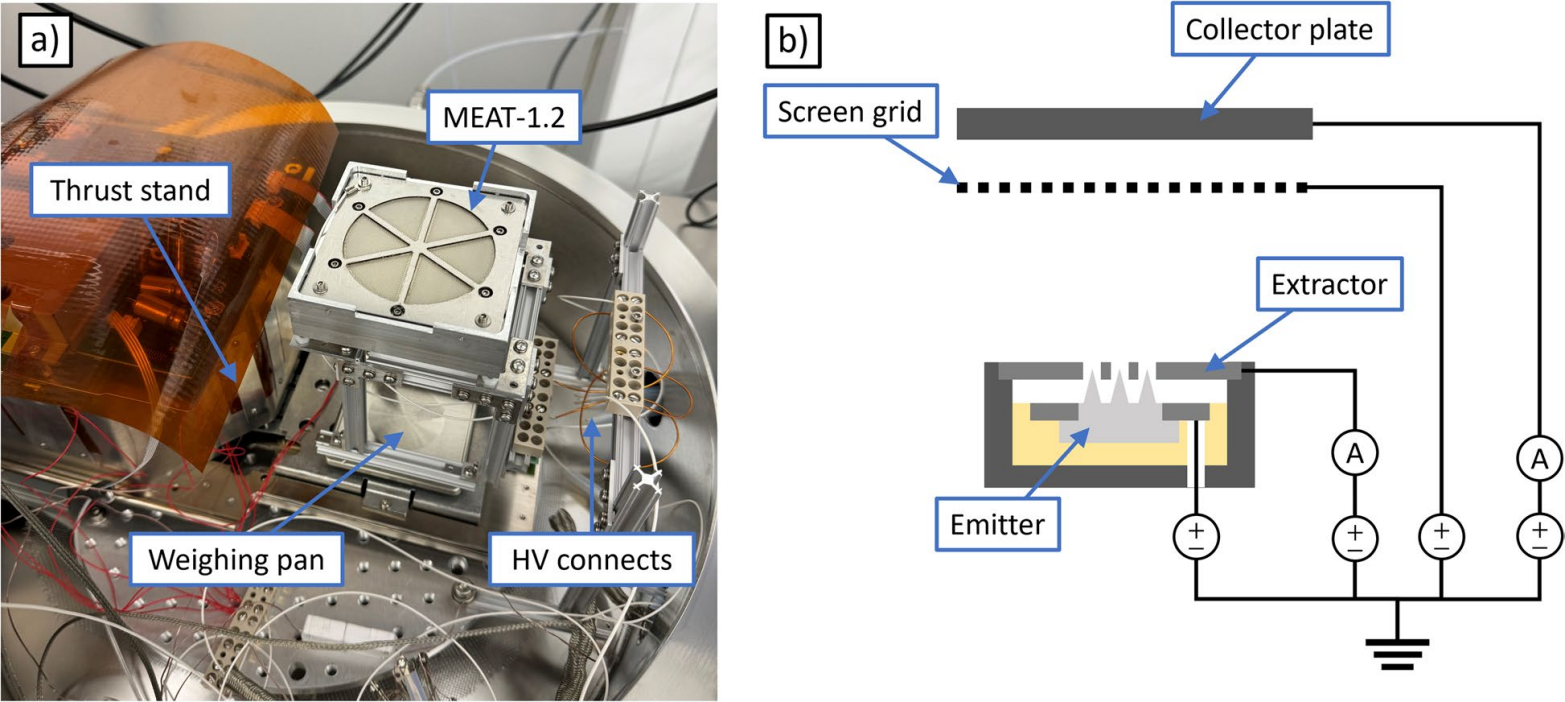
Bell jar experimental setup: **a** photograph and **b** electrical schematic

The first facility we utilized was MPL’s Bell Jar 1, a 0.4 $\times$ 0.6 m stainless steel bell jar which achieves high vacuum through a turbomolecular pump. For our experiments, the base pressure in the facility was 1.2 $\upmu$Torr. The key apparatus in the facility is a micronewton thrust stand, consisting of an analytical mass balance (Mettler-Toledo AX504) adapted to be vacuum-compatible. The mass balance has a capacity of 510 g, a readability and repeatability of 0.1 mg each, and a linearity of +/- 0.4 mg. The thruster was mounted face up on a support frame, resting wholly on the weighing pan of the mass balance; we show a photograph in Fig. 4a.

In this way, during operation the thrust stand yielded both the thrust of the system and its change in mass over time, which can be used to directly compute specific impulse and efficiency.

Since the extractor is tied electrically to the main thruster housing, PEEK screws were used to isolate the thruster body from its support frame. Electrical connections for the emitter and extractor were routed off the weighing pan by coiled wires (right side of Fig. 4a) designed to reduce spring force from the wires on the mass balance. Further, this spring force had negligible influence on the measurement because the mass balance operates in a null displacement mode; this insensitivity was verified when the thruster was fully installed by placing a series of calibration masses from 1 to 100 mg on the weighing pan to ensure they indicated the correct mass change within the readability of the balance. Additionally, a series of heaters attached to the mass balance maintained it an internal temperature of 305 K to mitigate thermal drifts.

Figure 4b is an electrical schematic of the test set up. The emitter and extractor voltages were set independently, with the emitter controlled by a reversible-polarity high voltage power supply (Matsusada EJ-2R100) and the extractor controlled by a 4-quadrant power supply (Kepco BOP 1000M). The voltage and current sourced to the emitter and the voltage sourced to the extractor were monitored internally by their respective power supplies. We measured the extractor current using a digital multimeter.

We suspended a beam dump above the thruster to discourage secondary charge flux to the system. The beam dump consists of a 20 $\times$ 20 cm stainless steel plate with an isolated mesh of tungsten wires in front. The thruster sat 10 cm below and—due to limited space around the bulk of the thrust stand—approximately 5 cm off center, such that the beam dump subtended a half angle of 30 degrees to one side and 60 degrees to the other, relative to thruster center. The screen grid and collector plate were biased with separate power supplies, and the collector current is measured by a digital multimeter.

**Fig. 5:**
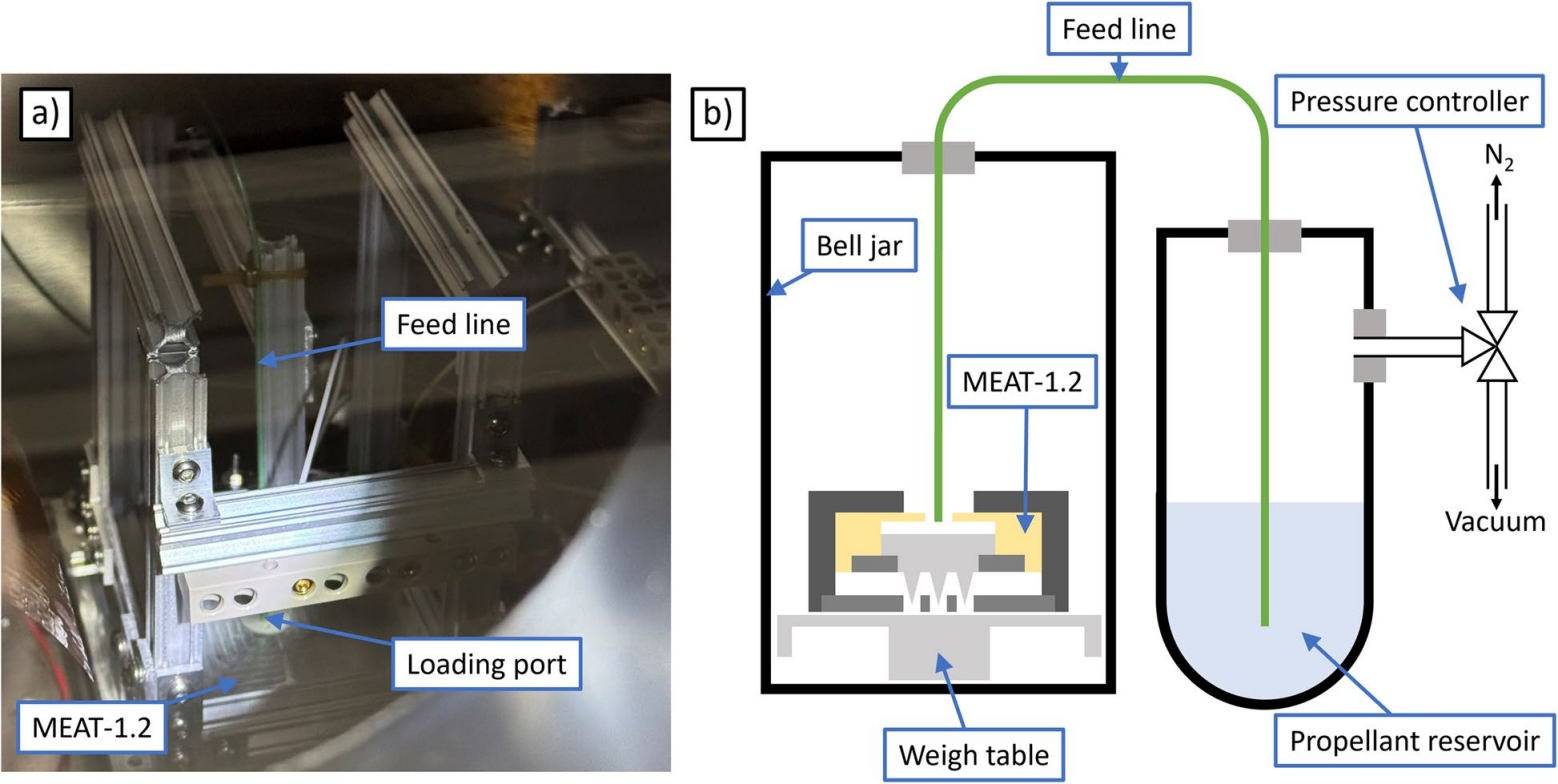
Bell jar loading configuration: **a** photograph of vacuum side and **b** schematic

Prior to our experiments, we configured the bell jar to load the thruster with propellant. The thruster was placed face down on the mass balance to expose a feed port on the underside of the emitter module. A PEEK tube was run from this port and fed through the bell jar to an external propellant reservoir. We include a photograph of the chamber side of this setup in Fig. 5a, and provide a schematic of the entire system in Fig. 5b. The reservoir can either be connected to vacuum or pressurized with nitrogen gas to force fluid through the tube and into the thruster. The reservoir includes a magnetic stir rod to accelerate degassing of the propellant at vacuum. The propellant line was removed from the bell jar after loading, necessitating a vacuum break.

#### 2 meter chamber

**Fig. 6:**
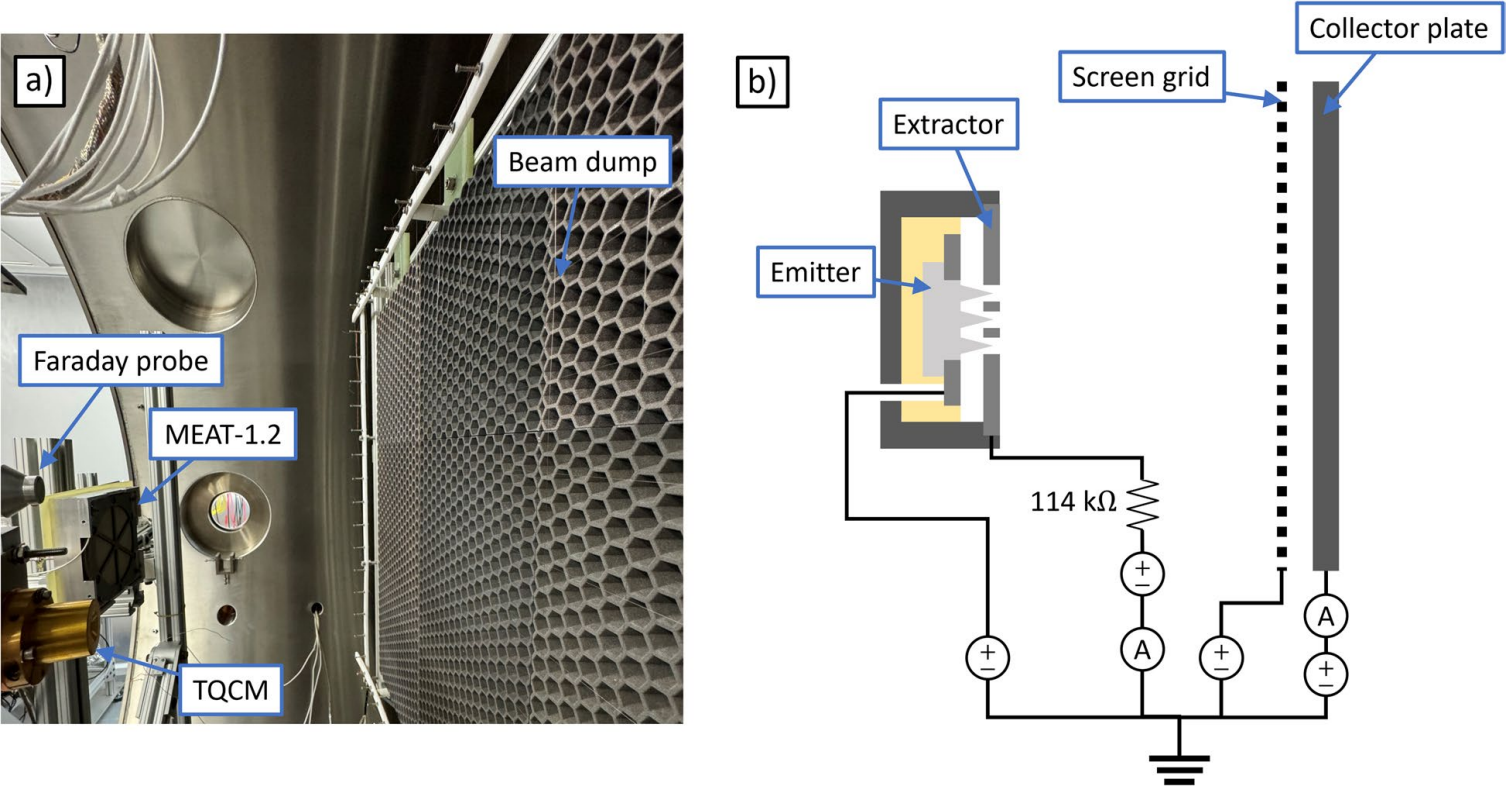
2 meter chamber experimental setup: **a** photograph and **b** electrical schematic

We performed additional experiments in MPL’s 2 meter chamber, a 2 $\times$ 2.5 m steel chamber that achieves high vacuum via cryopumping. The facility base pressure for our setup was 1.7 $\upmu$Torr. We show a photograph of the experimental configuration in Fig. 6a.

We mounted the thruster in a fixed position near the centerline of the chamber upstream of a beam dump. The beam dump consists of a 70 $\times$ 70 cm porous aluminum collector plate that is textured to recapture secondary species; a grid of tungsten wires is positioned in front of the plate. The beam dump was centered on the thruster and 30 cm downstream, such that it subtended a minimum half angle of 50 degrees relative to thruster center. Additionally, in order to measure secondary charge and mass flux from the beam dump, we positioned a Faraday probe and thermoelectric quartz crystal microbalance (TQCM) 10 cm to the side of the thruster, flush with its front plane, and oriented toward where the central axis of the thruster met the beam dump. For an expanded discussion of the beam dump design and performance, we refer to [20].

The electrical configuration for experiments in the 2 meter chamber (shown in Fig. 6b) was similar to that in the bell jar, with the following exceptions. First, rather than use a digital multimeter to sense the extractor current, we isolated the extractor supply and connected its return terminal to ground with a picoammeter (Keithley 485). To protect the ammeter against arcs and shorts between the electrodes, we placed a 114 kOhm resistor between the extractor and its power supply. The inclusion of this resistor created a modest (order 10 volts at peak power) voltage drop between the extractor and its supply as a result of intercepted current. Second, we replaced the beam dump collector power supply and digital multimeter with a source measure unit (Keithley 6450) to bias it and resolve the collected current simultaneously.

## Results

In this section, we present the results of our experiments, including details about the assembly and loading of the thruster, direct performance measurements made in the the bell jar facility, and more expansive throttling experiments done with the thruster in the 2 meter facility.

### Thruster alignment and loading

After assembling the thruster, we aligned the extractor and emitter electrodes using an optical microscope with a 3 axis position readout. We first roughly aligned the two laterally to provide clearance for the emitters, and then we adjusted the height of the emitters to provide the desired separation with the extractor. We subsequently realigned the emitters laterally to center them in their respective apertures. This alignment was conducted over a 5 point stencil consisting of 4 emitters at the periphery of the chip (in relative north, south, east, and west positions) and one emitter toward the center of the chip. We summarize the final alignment over this stencil in Table 1. Basal recession refers to the distance between the emitter basal plane and the metallized surface of the extractor. Emitter recession is the distance from the metallized face to the tip of an emitter, which we have computed assuming an emitter height of 455 $\upmu$m; practically, the emitters will be shorter as a result of manufacturing tolerances, so this estimate serves as a lower bound. Finally, the lateral misalignment is the distance between the center of the emitter and the center of the aperture parallel to the basal plane.

**Table 1:** Relative alignment of emitter and extractor electrodes over a 5 point stencil

Location	Basal recession	Min. emitter recession	Lateral misalignment
North	544 $\upmu$m	89 $\upmu$m	8.9 $\upmu$m
South	546 $\upmu$m	91 $\upmu$m	8.7 $\upmu$m
East	595 $\upmu$m	140 $\upmu$m	0.8 $\upmu$m
West	593 $\upmu$m	138 $\upmu$m	6.5 $\upmu$m
Center	484 $\upmu$m	29 $\upmu$m	6.6 $\upmu$m

We find that we were not able to adjust the height and tilt of the emitter module such that the basal recession was uniform across the emitter chip. This suggests the extractor electrode was warped, such that its center sits 60–120 $\upmu$m below its periphery. The warping is less along the north-south axis than the east-west, which may be because the north-south axis is directly aligned with one of the support struts (see Fig. 1). Given this warp, the final alignment of Table 1 was chosen to minimize the emitter recession over the chip while maintaining a vacuum gap (27 $\upmu$m) between the emitter basal plane and the underside of the extractor at the chip center to prevent fluid transport from the emitter. Otherwise, the lateral misalignment of the emitters was within 9 $\upmu$m, having minimal effect on emission.

After aligning the thruster, we mounted it in its support bracket and transferred it to the bell jar facility for propellant loading. The reservoir was filled with 1-ethyl-3-methylimidazolium bis(trifluorosulfonyl)imide (EMIM TFSI) and the entire system brought to high vacuum (<2 $\upmu$Torr) to degas the propellant overnight. After degassing, 18 g of propellant was infused into the emitter module. This 18 g target, about 12 mL volume, was chosen to be sufficient to fully saturate the emitter chip (7 mL volume) while only partially filling the reservoir (8.5 mL volume). The first 17 g were infused at a rate of 3 mg/s, while the final 1 g was infused at a rate of 1 mg/s. After loading, we allowed the thruster rest overnight at high vacuum to ensure the propellant was fully redistributed within the porous media of the emitter module.

### Direct performance measurements

Having assembled and loaded the thruster, we changed from the loading configuration of the bell jar to the experimental configuration, constituting a 90 min. vacuum break. We subsequently restored vacuum and allowed the thruster to degas overnight (20 hours), proceeding with experiments thereafter. We operated the thruster in a negative polarity mode, biasing the emitter below ground, $V_{\textrm{em}}<0$, while keeping the extractor grounded, $V_{\textrm{ex}}=0$. The beam target collector and screen were biased positively—to $V_{\textrm{c}}=+100$ V and $V_{\textrm{s}}=+200$ V, respectively—so the beam target would preferentially collect negative secondary species and the screen would provide a bias to retain positive secondary species. We gradually increased the magnitude of the emitter voltage until we were able to observe spray via the current monitor on the emitter power supply, which occurred at approximately −500 V, sourcing an emitter current, $I_{\textrm{em}}$, of order $-5\ \upmu$A.

We continued to increase the emitter voltage to condition the thruster for operation. At −1000 V, the emission current began to increase with time, from $-52\ \upmu$A to $-63\ \upmu$A over a period of 20 s. Increasing $V_{\textrm{em}}$ to $-1100$ V, we saw $I_{\textrm{em}}$ grow more dramatically, from $-120\ \upmu$A to $-573\ \upmu$A over a 70 s period. Thereafter, however, the emission current ceased to increase over time, so we proceeded to take performance measurements.

**Fig. 7:**
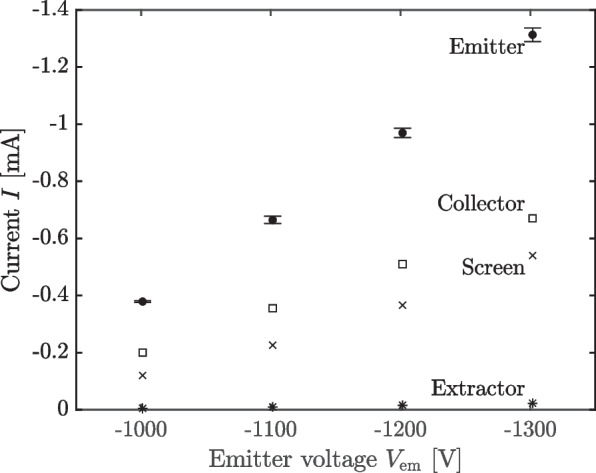
Currents as a function of emitter voltage for direct performance measurements in the bell jar facility

To conduct our measurements, we turned on the source for 50–90 s at a time beginning at $V_{\textrm{em}}=-1000$ V and incrementing by 100 V, turning off the thruster for at least 60 s in between set points. In Fig. 7, we plot the current sourced by the emitter, $I_{\textrm{em}}$, and the currents collected by the extractor, beam target collector, and beam target screen—$I_{\textrm{ex}}$, $I_{\textrm{c}}$, and $I_{\textrm{s}}$, respectively—as a function of emitter voltage.

We found that at $-1100$ volts and above, the emitter current exhibited a transient spike on starting up, which would subside over 20–30 s and settle into a slower, steadier decay in current with time. This kind of emission transient has been observed previously in electrospray sources [21–24], and is generally attributed to the fluidic properties of the source—i.e., a surfeit of propellant on the surface allows the emitter to source higher currents temporarily, before being throttled by its impedance. For our purposes, we exclude the period corresponding to this spike from our measurements. To obtain the emitter current data of Fig. 7, then, we smooth the current data over the remaining time using a LOESS method to remove noise from the monitor. The data in Fig. 7 are then the mean of these smoothed current curves, while the error, $\sigma _{I_{\textrm{em}}}$, is taken as the standard deviation. The error bars correspond to a 95% interval ($\pm 1.96\sigma$). The collector, screen, and extractor currents were recorded manually from their instruments as single measurements and so lack estimates for their error.

We observe that the emitter, collector, screen, and extractor current are essentially linear over this domain. The collector and extractor current are also nearly a constant proportion of the emitter current, approximately 52% and 1.5%, respectively. The screen current is not a constant proportion, instead increasing from 32% to 41% monotonically as $V_{\textrm{em}}$ increases. That the collector and screen currents are of comparable order is surprising, considering that, geometrically, the screen subtends orders of magnitude less of the beam than does the collector. This suggests the screen bias is acting to attract and collect a comparatively large population of negative secondary species, such as electrons liberated from the collector. These negative secondaries reduce the current read on the collector relative to the incident charge flux, and so the most robust estimate of the total beam current captured by the beam target is the sum of the collector and screen currents. Together, the collector and screen currents indicate the beam target captures between 85–95% of the incident beam current.

**Fig. 8:**
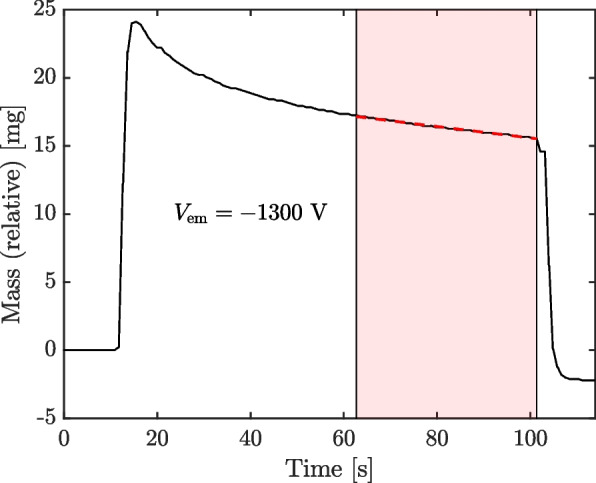
Example raw mass trace (black) and linear fit to calculate mass flow rate (dashed red)

Using the mass balance, we were able to obtain measurements of thrust, *T*, and mass flow rate, $\dot{m}$, over these same windows. Using the current and voltage data of Fig. 7, we can also compute the emission power, *P*, the specific impulse, $I_{\textrm{sp}}$, and the thruster efficiency, $\eta$:

1a $$\begin{aligned} P & = I_{\textrm{em}}V_{\textrm{em}}, \end{aligned}$$

1b $$\begin{aligned} I_{\textrm{sp}} & = \frac{T}{g_0\dot{m}}, \end{aligned}$$

1c $$\begin{aligned} \eta & = \frac{T^2}{2\dot{m}P} , \end{aligned}$$

where $g_0$ is the standard gravity. The mass balance measures the thrust and weight of the system simultaneously, producing a mass reading trace exemplified by Fig. 8, which is for $V_{\textrm{em}}=-1300$ V.

There is an initial step up when the thruster is turned on, followed by an inflected region corresponding to the current transients just after startup, then a steady region of decreasing mass corresponding to the mass flow rate, and finally another step down when the thruster is shut off. To calculate mass flow rate, for each setpoint we fit a line (by least squares) to the region immediately preceding turning off the thruster, the same windows as were used to compute the emitter current data of Fig. 7. To estimate the error in the mass flow rate, $\sigma _{\dot{m}}$, we use the error bounds for the slope parameter returned from the curve fitting algorithm (see [25]). We render this fit in Fig. 8 as a dashed red line and shade the interval over which the fit was performed. To determine the thrust, we take the difference between the mass reading when the thruster is shut off to after it has settled (compare to the tail end of Fig. 8); the error in thrust, $\sigma _T$, we estimate as one-half the readability of the mass balance, or 50 $\upmu$g. Finally, we estimate the error for the derived quantities of Eqs. 1 through conventional error propagation, wherein each equation is approximated by a first-order Taylor series expansion about the datum and the variance of each uncertain parameter propagated assuming they are uncorrelated,

2a $$\begin{aligned} \sigma _P & \approx \sqrt{\left( V\right) ^2\sigma _I^2}, \end{aligned}$$

2b $$\begin{aligned} \sigma _{I_{\textrm{sp}}} & \approx \sqrt{\left( \frac{1}{g_0\dot{m}}\right) ^2\sigma _T^2 + \left( \frac{T}{g_0\dot{m}^2}\right) ^2\sigma _{\dot{m}}^2}, \end{aligned}$$

2c $$\begin{aligned} \sigma _\eta & \approx \sqrt{\left( \frac{T}{\dot{m}P}\right) ^2\sigma _T^2 + \left( \frac{T^2}{2\dot{m}P^2}\right) \sigma _P^2 + \left( \frac{T^2}{2\dot{m}^2P}\right) \sigma _{\dot{m}}^2}, \end{aligned}$$

where we have neglected the minimal error in the voltage data.

**Fig. 9:**
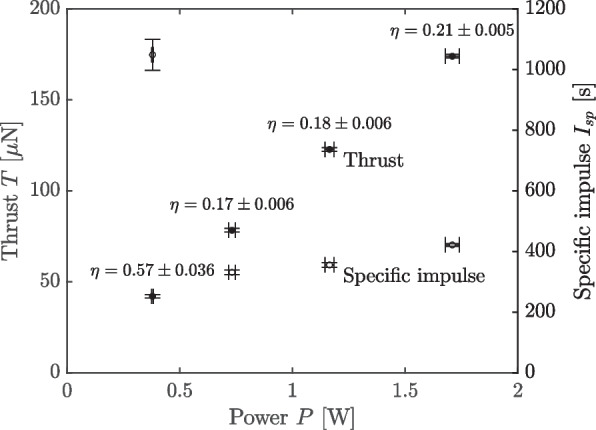
Thrust, specific impulse, and efficiency as a function of power for bell jar experiments

We plot the thrust and specific impulse as a function of power in Fig. 9. We also include the thruster efficiency computed at each power level.

The error bars correspond to a 95% confidence interval. We find that the thrust increased nearly linearly with power, demonstrating a maximum of 174 $\upmu$N at 1.7 W. In contrast, the specific impulse, and hence the efficiency, drop precipitously between the $-1000$ V and $-1100$ V setpoints and begin to recover gradually thereafter, with a peak specific impulse of 1050 s and efficiency of 57%. That this drop in efficiency occurs while the thrust to power ratio, $\frac{T}{P}$, remains nearly constant suggests that the thruster is shedding mass that does not enter the beam, a point we return to in the discussion (see Efficiency loss at higher power).

We note finally that the thruster power for these experiments was limited by the pressure in the facility. In attempting to operate the thruster at $V_{\textrm{em}}=-1400$ V, the facility pressure rose to order 200 $\upmu$Torr within one second. An arc from the distal electrode to the thruster body then disabled the mass balance, requiring the thruster to be removed to reinitialize it. Subsequent testing without telemetry from the mass balance revealed that the facility pressure over the domain $V_{\textrm{em}}=[-800,-1100]$ was a nearly linear function of the emission current, varying from approximately 7 $\upmu$Torr at $-125\ \upmu$A to 42.8 $\upmu$Torr at $-680\ \upmu$A.

### Expanded throttling

**Fig. 10:**
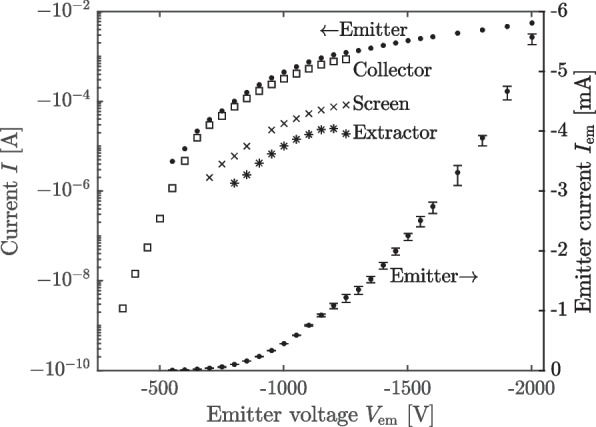
Currents as a function of emitter voltage for initial 2 meter chamber negative polarity throttling

To throttle the thruster over a wider power range, we conducted additional experiments in the 2 meter facility at NASA JPL MPL, which has approximately a factor 10 higher pumping speed than the bell jar. After transferring the thruster to the new facility and allowing it to acclimate overnight, we first gradually increased voltage in a negative polarity mode, from $V_{\textrm{em}}=-300$ to $V_{\textrm{em}}=-2000$, while keeping the extractor grounded. Each voltage condition was maintained for 20–90 s, without turning the thruster off in between. The 2 meter chamber beam dump was biased similarly to that in the bell jar, $V_{\textrm{s}}=+200$ and $V_{\textrm{c}}=+100$. We show the results of this sweep in power in Fig. 10.

We render the emitter, collector, screen, and extractor currents first on a logarithmic scale to capture the high dynamic range of these quantities over the full sweep. We then re-render the emitter current data on a linear scale for comparison.

We first observed emission at $-350$ V, extracting a current of order nA ($\sim 1\ \upmu$W), as seen from the beam target collector. While the current monitor of the emitter power supply was not capable of resolving this small current, it was discernible using the source measure unit connected to the collector. The peak emission current was $-5.6$ mA at $-2000$ V (11.2 W), corresponding to an order 10^7^ variation in power. We find that, where measured, the collector, screen, and extractor currents are each almost a constant proportion of the emitter current, 69%, 6.5% and 2.0%, respectively. The facility pressure again varies approximately linearly with emitter current, from 2 $\upmu$Torr at $-750$ V and $-6\ \upmu$A to 31 $\upmu$Torr at $-2000$ V and $-5.6$ mA.

The emitter current exhibits a characteristic inflection in log space, growing strongly below $-600$ volts, exhibiting a knee between $-600$ and $-1300$ V, and increasing more weakly above $-1300$ volts. These exponential growth rates mean that in linear space the current is inflected over the entire domain, with the slope $\frac{\textrm{d}I_{\textrm{em}}}{\textrm{d}V_{\textrm{em}}}$ increasing from about 7 nA/V to 9 $\upmu$A/V. This type of inflection is often observed at both individual emitter [7, 9, 26–30] and array [7–9, 11, 31–33] scale in porous sources, and it arises as a complex interaction of emitter variability in the array [9, 31, 34] and individual emitter physics [17, 35]. Due to this variability, additional emitters become active as the voltage increases. Indeed, at the very lowest voltages where we measured emission current, it is likely that only a few emitters among the 6102 present are active.

We observed, as was the case in the bell jar, that at $-1200$ V and above there were transient spikes in current whenever the voltage was changed, which became more pronounced as the emitter voltage was increased. In contrast to the bell jar experiments, however, we saw that, for all voltages where the emitter current was measurable via its monitor, the emitter current exhibited a decay in current over time. Based on this strongly time-varying behavior, the emitter current data of Fig. 10 represent the mean of the smoothed current trace over that setpoint, while the vertical error bars represent the maximum and minimum of the smoothed current over the same interval. The high variability in the higher voltage data is the result of not allowing the current to fully settle before transitioning to the next set point, which we did to limit the amount of time the source was operated at higher voltages where the longer scale emission decay appeared more pronounced.

### Positive mode and repeatability

**Fig. 11:**
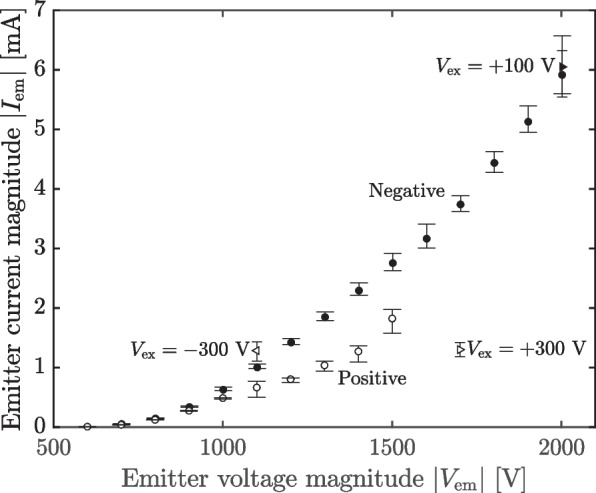
Positive and negative polarity emitter current measurements for repeatability tests in 2 meter chamber

For comparison and to examine the repeatability of the thruster, we operated in a positive emission mode, sweeping up the emitter voltage with the extractor grounded as before. The thruster was turned off or operated only intermittently at low voltages ($<1000$ V) for 42 h before performing the sweep. The beam target polarity was also inverted, such that $V_{\textrm{s}}=-200$ and $V_{\textrm{c}}=-100$. The emitter current as a function of emitter bias appears in Fig. 11 (open circles), where the current data were processed as in the Expanded throttling section.

We found in raising the emitter voltage from 600 V to 1000 V that the current did not exhibit transients on startup or any emission decay. Beginning at 1100 V, however, these features manifested consistent with negative mode testing in the Expanded throttling section. Additionally, we observed that below 1100 V the emission current in positive mode is comparable or larger in magnitude than the emission current in negative mode for the same voltage, but that at and above 1100 V the positive mode sources decidedly less current for comparable voltage.

We also saw that when increasing to 1400 V from 1300 V, the emission current signal became noisier, from a root mean square 5% of the mean to 10% of the mean. Upon inspection, we determined this was the result of small arcs forming across the thruster. Anticipating that these arcs might be caused by secondary particles streaming back to the emitters from the facility, we attempted to suppress the arcs by modifying the extractor bias, $V_{\textrm{ex}}$, to be $\pm 300$ V while maintaining the same extraction potential, $V_{\textrm{em}}-V_{\textrm{ex}}=\Delta V$. We also plot the corresponding data in Fig. 11 (forward and backward triangles). The thruster sourced comparable currents at these alternative operating points, but neither succeeded in eliminating the arcs. We thus terminated the sweep after confirming that the arcs persisted at higher voltages.

To examine the repeatability of the thruster in negative mode after this less stable operation, we performed an additional sweep in negative polarity, which we render alongside the positive mode data in Fig. 11 (closed circles). The beam dump was again set to $(V_{\textrm{c}},V_{\textrm{s}})=(+100,+200)$. Qualitatively, this sweep is similar to that of Fig. 10; however, quantitatively, we observe that the emitter current is higher than previously. This discrepancy is of order 10 $\upmu$A at $-700$ V, growing to 500 $\upmu$A at $-1300$ V and remaining of comparable order thereafter. Higher emitter currents could be explained by potential leakage from the emitter to the extractor (i.e., having formed during the arcing in positive mode). Though a software error means we lack extractor current measurements for these data and so cannot directly assess this possibility, the nonlinear behavior exhibited suggests leakage is not the cause. Additionally, when performing a spot check of the collector current at $-1400$ V, we found that it was still approximately 70% of the emitter current, indicating that the same proportion of the emitter current is making its way into the beam. It is likely, then, that this increase in emission current is caused by another process, such as modification of the local emitter geometry by the formation of carbonized protrusions [36].

Because the emitter supply is limited to $+2000$ V, to explore higher power operation we also include one datum with the extractor set to $V_{\textrm{ex}}=+100$ V (filled forward triangle). As a result of the time-dependent behavior of the source, the peak current at this setpoint was lower than that of the $(V_{\textrm{em}},V_{\textrm{ex}})=(-2000,0)$ V set point, though its average current is higher. The peak power was thus 13.3 W, with a mean of 12.7 W.

### Time-dependent behavior

**Fig. 12:**
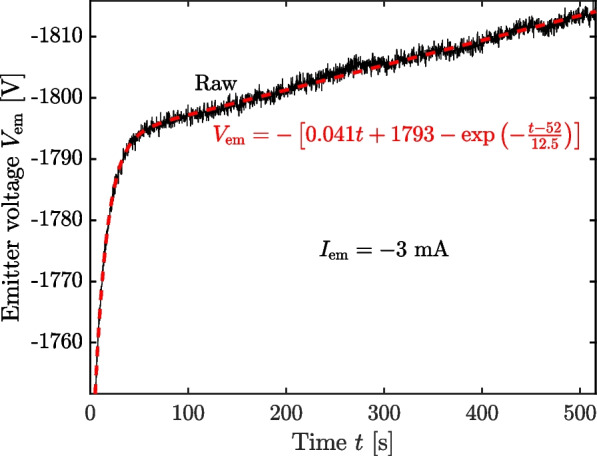
Emitter voltage trace for current-controlled operation at $I_{\textrm{em}}=-3$ mA

To characterize long scale changes in performance, we turned on the thruster for an extended period of time. We operated the thruster for 516 s in a current-controlled negative polarity, with $I_{\textrm{em}}=-3$ mA and the beam dump biased to $(V_{\textrm{c}},V_{\textrm{s}})=(+100,+200)$. Figure 12 shows the resulting trace of emitter voltage over time (black line).

Mirroring the transients in current seen in voltage-controlled operation, the emitter current rises quickly when the thruster is turned on, converging to a linear increase in voltage with time after approximately 50 s, indicating a longer time scale decay in emission. We model this time dependency as an exponential rise superposed with a line:

3 $$\begin{aligned} V_{\textrm{em}} = -\left[ mt+b-\textrm{exp}\left( -\frac{t-t_0}{\tau }\right) \right] , \end{aligned}$$

where *m* and *b* are the slope and offset of the linear background, $\tau$ is the time constant of the exponential rise, and $t_0$ is a scale parameter indicating the time at which the exponential component is unity. The negative sign in front of the right hand side indicates negative polarity. We show a least squares fit of Eq. (3) to the data in Fig. 12 (red dashed), along with the corresponding best-fit parameters.

The model captures the data well on both the comparatively short time scale dominated by the exponential rise and the longer time scale defined by the steady increase in voltage with time. Consistent with earlier measurements in voltage-controlled mode, we observe a time constant of $\tau =$ 12.5 s. We note that this time constant is long compared to that of individual emitters (often only a few ms) [21, 23, 24], but is more consistent with those of larger-scale thrusters [37–39], suggesting the fluidic phenomena controlling flow scale with the size of the system. The slew rate of $m=$ 41 mV/s is comparatively rapid, causing the power required to maintain emission current to increase by 0.9% from $t=100$ s to $t=500$ s. Indeed, at this rate, the 2 kV limit of the power supply would be reached after only 75 min. This long-scale decay in emission current is frequently observed in porous electrosprays [32, 38, 40] and is attributed to a variety of mechanisms, including electrochemical degradation of electrodes [41, 42], depletion of porous reservoirs [7, 43], or degradation from backstreaming facility secondary species [44]. While all of these mechanisms likely contribute to the observed decay, backstreaming secondaries are of particular interest as a facility effect, a point we examine in the discussion.

## Discussion

In this section, we discuss our results, interpreting them physically and placing them within the context of continued thruster development.

### Efficiency loss at higher power

We found in performing direct performance measurements of the MEAT-1.2 that there was a sudden decrease in efficiency between $-1000$ V and $-1100$ V operation, from 57% to 17%. This was accompanied by a concomitant decrease in the specific impulse, from 1050 s to 330 s. When we compute the mean specific charge of the beam, $\left\langle \xi \right\rangle$, from our measurements of emitter current and mass flow rate,

4 $$\begin{aligned} \left\langle \xi \right\rangle =\frac{I_{\textrm{em}}}{\dot{m}}, \end{aligned}$$

we find that $\left\langle \xi \right\rangle$ falls from $-93$ C/g to $-28$ C/g. For the EMIM TFSI propellant used here, that corresponds to a mean coordination number of approximately 2 (i.e., a trimer) and 8, respectively. Recognizing that the spray is in reality polydisperse, this would suggest that at $-1000$ V the beam is primarily ionic, while at $-1100$ V and above the thruster begins also to emit higher mass species, such as high solvation state clusters or droplets.

However, when we compare against our thrust data (see Fig. 9), we see that the thrust is nearly a linear function of power; that is, the thrust to power ratio, $\frac{T}{P}$, is nearly constant. If it were the case that higher-mass species were being produced by the thruster and accelerated in the beam, we would expect the thrust to power ratio to increase, which we can intuit by modeling a beam of specific charge $\xi$. We idealize the beam as perfectly collimated and monodisperse, and we assume the particles are accelerated by the entire potential *V*. They then reach a velocity

5 $$\begin{aligned} v=\sqrt{2\xi V}, \end{aligned}$$

produce a thrust of

6 $$\begin{aligned} T=\dot{m}v=I\sqrt{\frac{2V}{\xi }}, \end{aligned}$$

and hence a thrust to power of

7 $$\begin{aligned} \frac{T}{P}=\sqrt{\frac{2}{\xi V}}. \end{aligned}$$

**Fig. 13:**
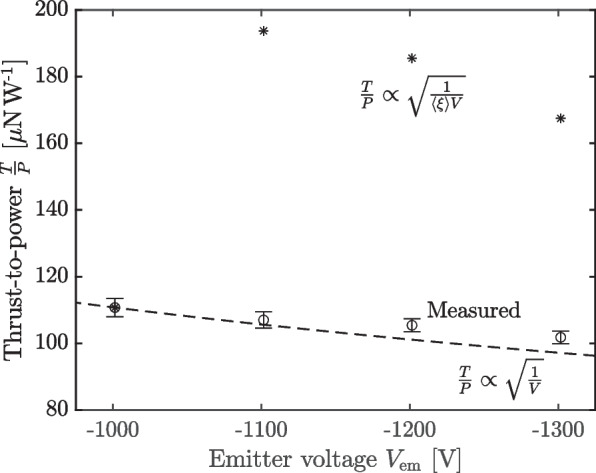
Thrust to power ratio for bell jar experiments, measured (circles), assuming scaling only with voltage (dashed line), and assuming scaling with voltage and derived charge to mass (*)

We contrast this anticipated $\frac{T}{P}$ scaling against our bell jar experiments in Fig. 13, where the black circles are the experimental measurements.

The error bars follow the analysis of Eqs. (2),

8 $$\begin{aligned} \sigma _{\frac{T}{P}}\approx \sqrt{\left( \frac{1}{P}\right) ^2\sigma _T^2 + \left( \frac{T}{P^2}\right) ^2\sigma _P^2}, \end{aligned}$$

where again we plot a 95% confidence interval. We then take the −1000 V measurements as a reference condition from which to compute the scaling of Eq. (7), where we substitute the average charge to mass ratio computed from our current and mass flow rate measurements $\left\langle \xi \right\rangle$, Eq. (8), for the idealized monodisperse charge to mass ratio, $\xi$. These points are denoted by asterisks, and we observe that after the transition from −1000 V to −1100 V they overestimate the observed thrust to power. Acknowledging that this is only a rough approximation because we have not accounted for polydispersity, we nevertheless observe that the scaling of Eq. (7) is a poor explanatory model for the data, with this projection lying far outside our confidence in the measurement.

As an alternative hypothesis, and again using the −1000 V condition as a reference point, we compute what thrust to power we would expect at higher voltages assuming the charge to mass ratio $\xi$ was the same as for the −1000 V case, such that $\frac{T}{P}\propto \sqrt{\frac{1}{V}}$ only (the dashed line in Fig. 13). We see that this projection much more closely tracks the data. We infer, therefore, that this second hypothesis—that the charge to mass ratio of the beam does not vary with voltage compared to the −1000 V condition—is much more plausible given the data. This means, then, that the thruster must be losing mass by a means other than it being coupled into the beam. That is, if there do exist massive species that are being ejected from the thruster, they must be at very low energies or with extremely high divergence.

This “anomalous mass loss”, whereby a thruster loses mass that does not appear to be ejected in its beam, has been observed previously, and may be the result of electrochemistry at the distal electrode, thermal evaporation of the propellant, and decomposition of the propellant by high energy particles, the latter two of which can be caused by backstreaming facility secondary species [44, 45]. Charged particle flux back to the thruster is especially likely an explanation. Measurements taken with the reverse Faraday probe (see [20]) verify a flux of backstreaming charged particles, and we observed that the thruster body was discolored after testing, suggesting decomposed propellant or other ejecta had condensed on its surface. Thus, there was a population of free charged species that could bombard the emitters and induce heating or propellant decomposition.

Secondary charged species are also likely responsible for the arcing we observed in positive mode. That this phenomenon only occurs in positive polarity suggests that it is contingent on the asymmetry in mobility of positive and negative secondaries—i.e., that in positive mode electrons are preferentially attracted to the emitters to break down propellant and induce arcs. We were also unable to deter these arcs by adopting a negative potential on the extractor, suggesting at least two possibilities: 1. We applied insufficient bias. The extractor (see Fig. 3) does not perfectly shield the emitters, and the negative beam dump bias increases the voltage necessary to reject escaped secondaries. 2. Ionic current to the extractor—whether from intercepted primary beam ions or backstreaming secondary ions—produces additional secondary electrons and anions that are then strongly attracted to the emitters. In any case, our observations warrant additional experimentation and highlight the continued importance of facility effects in electrospray thruster testing, particularly the role of secondary species [46, 47].

### Implications for scalability

The construction of the MEAT-1.2 represents an order of magnitude increase in the number of emitters fabricated in a single chip compared to previous porous conical type systems, a capability enabled by the manufacturing strategies adopted in the Thruster design and manufacturing section. While experimental limitations prevented us from directly characterizing performance at elevated powers, even the performance demonstrated at lower power MEAT-1.2 operation (42 to 174 $\upmu$N at $\sim$100 $\upmu$N/W and 57 to 21% efficiency) compare favorably with many other porous systems [7, 8, 32, 38, 43]. Further, if we assume a thrust to power equal to the highest voltage condition of Fig. 9 (102 $\upmu$N/W) and extrapolate to the highest power condition of Fig. 11 (12.7 W), we would expect a peak thrust of 1 mN (about 165 nN per emitter), a peak thrust density of 130 mN/m^2^, and minimum specific power of 25 kg/kW. This projected thrust density in particular highlights the advantage of adopting a monolithic scaling strategy to minimize parasitic support structure; for comparison, the AFET-2, from which the MEAT-1.2 most directly draws its design heritage, demonstrated a maximum thrust density of 38 mN/m^2^[8], a potential factor 4.6 increase. This thrust density is still far below that of state of the art Hall thrusters (order 1–10 N/m^2^), however, so while electrosprays represent an efficient propulsive solution at low power, competitiveness at higher powers is still limited by emitter packing density [1].

Further work is warranted, however, to understand the robustness of the MEAT-1.2 at scale. We have not yet been able to thoroughly characterize tolerances in manufacturing and alignment (cf. [15]), but the inflected current curve in Fig. 10 suggests that there exists nonnegligible variability between emitters. The warp in the extractor evident in Table 1 also means that alignment between the electrodes varied comparatively widely across the array. This inhomogeneity is likely detrimental to long-duration operation [12]. Additionally, we lack a detailed explanation for the long scale decay in emission current observed in both positive and negative polarity. This is likely at least partially a facility effect stemming from backstreaming secondary species and emphasizes an important experimental trade in pursuing a system of this scale: while the higher thrust and mass flow rates make resolving thruster performance much more directly accessible, they also tax the test facility and require enhanced controls.

## Conclusion

The design, manufacture, and initial characterization of the MEAT-1.2 we presented here constitutes a key component in robustly scaling porous electrosprays to higher powers. We showed via direct thrust measurements that a single chip system with an order of magnitude greater emitters achieved comparable performance to the state of the art. The 6102 emitters of the thruster achieved 174 $\upmu$N of thrust and 21% efficiency at 1.7 W, and in subsequent experiments we throttled the thruster up to 13.3 W peak power, at which by extrapolation we might anticipate 1 mN of thrust. The corresponding expected thrust density of 130 mN/m^2^ suggests that increasing system power by building larger chips is advantageous for minimizing system footprint. Further effort is needed to more fully characterize system performance, however, particularly over long time scales.

The experimental data collected here lend themselves to a robust model-based design optimization for the system. Future work will focus on more thoroughly characterizing variance in emitter geometry and on regressing predictive engineering models against experimental observations. Such analyses provide a rigorous uncertainty quantification backbone capable of considering the fundamentally probabilistic problem of emitter failure and thruster lifetime. Altogether, the MEAT-1.2 demonstrates a feasible architecture by which to achieve the orders of magnitude higher thruster and power needed for small spacecraft.

## Supplementary Information

Supplementary Material 1.

## Data Availability

The data used and generated in this study are available in the supplementary material.
